# Structural asymmetries of the human cerebellum in relation to cerebral cortical asymmetries and handedness

**DOI:** 10.1007/s00429-016-1295-9

**Published:** 2016-08-26

**Authors:** Tulya Kavaklioglu, Tulio Guadalupe, Marcel Zwiers, Andre F. Marquand, Marten Onnink, Elena Shumskaya, Han Brunner, Guillen Fernandez, Simon E. Fisher, Clyde Francks

**Affiliations:** 1 Language and Genetics Department, Max Planck Institute for Psycholinguistics, Nijmegen, The Netherlands; 2 International Max Planck Research School for Language Sciences, Max Planck Institute for Psycholinguistics, Nijmegen, The Netherlands; 3 Donders Center for Cognitive Neuroimaging, Donders Institute for Brain, Cognition and Behavior, Radboud University, Nijmegen, The Netherlands; 4 Department of Neuroimaging, Center for Neuroimaging Sciences, Institute of Psychiatry, King’s College London, London, UK; 5 Department of Human Genetics, Donders Institute for Brain, Cognition and Behavior, Radboud University Medical Center, Nijmegen, The Netherlands; 6 Donders Institute for Brain, Cognition and Behavior, Radboud University, 6500 Nijmegen, The Netherlands

**Keywords:** Cerebellum, Asymmetry, Language, Anatomical, Lateralization, Handedness

## Abstract

There is evidence that the human cerebellum is involved not only in motor control but also in other cognitive functions. Several studies have shown that language-related activation is lateralized toward the right cerebellar hemisphere in most people, in accordance with leftward cerebral cortical lateralization for language and a general contralaterality of cerebral–cerebellar activations. In terms of behavior, hand use elicits asymmetrical activation in the cerebellum, while hand preference is weakly associated with language lateralization. However, it is not known how, or whether, these functional relations are reflected in anatomy. We investigated volumetric gray matter asymmetries of cerebellar lobules in an MRI data set comprising 2226 subjects. We tested these cerebellar asymmetries for associations with handedness, and for correlations with cerebral cortical anatomical asymmetries of regions important for language or hand motor control, as defined by two different automated image analysis methods and brain atlases, and supplemented with extensive visual quality control. No significant associations of cerebellar asymmetries to handedness were found. Some significant associations of cerebellar lobular asymmetries to cerebral cortical asymmetries were found, but none of these correlations were greater than 0.14, and they were mostly method-/atlas-dependent. On the basis of this large and highly powered study, we conclude that there is no overt structural manifestation of cerebellar functional lateralization and connectivity, in respect of hand motor control or language laterality.

## Introduction

Left–right asymmetries are an important feature of the brains and behavior of humans (Toga and Thompson 2003). Left-hemisphere language dominance is one of the most prominently lateralized functional properties of the average human brain (Bethmann et al. 2007), while a strong population-level bias in hand preference (roughly 90 % right-handed) is a prominent behavioral lateralization (Hardyck and Petrinovich 1977). Most structural and functional studies of human brain laterality have focused on the cerebral cortex (Toga and Thompson 2003). Structural and functional lateralization have been observed throughout the cortical language regions surrounding the Sylvian fissure, including the pars opercularis and pars triangularis of the frontal lobe, and the superior temporal and transverse temporal regions of the temporal lobe (Toga and Thompson 2003). Hand preference is linked to functional lateralization for motor control around the precentral gyrus (Willems et al. 2014), while left-handedness has been tentatively linked with altered structural lateralization of this same cortical region (Amunts et al. 1996; Guadalupe et al. 2014). Furthermore, variations in language lateralization and hand preference are subtly related (Knecht et al. 2000; Mazoyer et al. 2014).

The cerebellum also shows functional lateralization, which has been best described in relation to motor control. Lateralized hand motor actions map to ipsilateral cerebellar lobules V and VIII with a high degree of precision (van der Zwaag et al. 2013). A similar ipsilateral relationship between motor actions and the posterior cerebellum was observed when cerebellar lobules VI, VIIb, and IX were electrically stimulated (Mottolese et al. 2013). However, there is evidence that the cerebellum is also involved in various cognitive processes in addition to motor control (Stoodley 2012). While anterior cerebellar lobules project extensively to contralateral cerebral cortical motor-related areas, the cerebellar hemispheres are also connected to predominantly contralateral cerebral cortical association networks via polysynaptic projections, including to prefrontal cortex (Buckner et al. 2011; Bostan et al. 2013; Buckner 2013). In fact, the cerebellum may support multiple and heterogeneous representations with respect to cerebral cortical regions (Manni and Petrosini 2004).

Language-related tasks are known to activate the cerebellum in a partly lateralized manner (Jansen et al. 2005; Lesage et al. 2015). The rightward lateralization of language-related activity in the cerebellum is consistent with left lateralized activation in cerebral cortical association regions (Petersen et al. 1989). More specifically, cerebellar lobules VI, Crus I, Crus II, and VIIb have consistently shown rightward lateralized language-related activation (Jansen et al. 2005; Stoodley and Schmahmann 2009; Filippi et al. 2011; Stoodley et al. 2012). This contralateral connectivity with the cerebral cortex manifests not only in task-dependent fMRI measurements but also in resting state activity (McAvoy et al. 2015). Furthermore, patients with cerebellar damage or developmental impairments often show both motor and cognitive disturbances (Schmahmann 1991; Ito 2008), and disorders, including dyslexia, autism, and specific language impairment (SLI), have been linked to altered functional activation patterns or structural asymmetry of the cerebellum (Baillieux et al. 2009; Hodge et al. 2010; Fernandez et al. 2013). In a study of 1000 subjects, cerebellar lobules VI, Crus I, and Crus II showed the strongest rightward lateralization of intrinsic brain activity (Wang et al. 2013). Lobule VI and the most anterior parts of Crus I and Crus II, as well as lobule VIII, showed the strongest leftward lateralization of intrinsic brain activity.

In contrast to lateralized cerebellar activation and its functional connectivity, relatively little is known about how structural asymmetries of the cerebellum may relate to structural asymmetries of language- and motor-related cerebral cortical regions, and to handedness. An overall rightwards volumetric asymmetry of the cerebellum was reported in a recent study conducted on 138 adults (Kang et al. 2015). In a study of 23 adults whose cerebellar images were divided into left–right and anterior–posterior segments, a global torque was described which differed by handedness (Snyder et al. 1995). At a regional level, an MRI study examining the morphometric differences between the left and right cerebellar lobules in 112 adults showed an overall rightward volumetric asymmetry, but a leftward asymmetry in medial posterior regions (Fan et al. 2010). The cerebellum also showed a left–right asymmetrical neurochemical organization in a study of postmortem tissue samples from 12 subjects, most of whom died due to cancer (Baizer 2014).

Here, we have used automated measurement of individual differences in volumetric asymmetries of cerebellar gray matter in 2226 healthy subjects, to test the correlations with structural asymmetries within language-related and motor-related cerebral cortical regions, and with handedness. For the cerebellum, we used a probabilistic atlas that parcellates the structure into its lobules. For the cerebral cortex, we defined language- and motor-related regions according to two different automated methods and cerebral cortical atlases. This was by far the largest study of cerebellar structural asymmetry to have been performed, as well as of its potential relations to cerebral cortical asymmetries and handedness.

## Methods

### Study data set

The brain imaging genetics (BIG) study was initiated in 2007 and comprises healthy volunteer subjects, including many university students, who participate in diverse imaging studies at the Donders Center for Cognitive Neuroimaging (DCCN), Nijmegen, The Netherlands (Franke et al. 2010). At the time of this study, the BIG subject-pool consisted of 2709 healthy adult volunteers (1435 females) who had undergone anatomical (T1-weighted) MRI scans, usually as part of their involvement in diverse small-scale studies at the DCCN, and who had given their consent to participate in BIG.

Handedness of the participants was assessed by an item in their enrolment form. This consisted of subjects selecting an answer from the two options “left-handed/right-handed” (in Dutch). Only those subjects who clearly indicated one or the other state were included in our analysis. This resulted in a sample of 2307 right-handed subjects and 119 left-handed subjects, with a mean age of 25.70 years and a standard deviation of 10.56 years. Note that the BIG study was not recruited to specifically study handedness, and therefore, only a simple binary measure was available. Nonetheless, simple self-assessments show close agreement with dichotomous scoring of handedness as derived from multi-item inventories (see “Discussion”). The proportion of left-handers was lower than in the general population; this was due to left-handedness being used as an exclusion criterion for some of the imaging studies that were pooled into the overall BIG dataset. Nonetheless, handedness was not associated with any particular acquisition protocol in the overall dataset (see below).

A subset of 381 subjects (345 right and 8 left-handed) had undergone a brain MRI scan twice, with at least 1-day separation between scans. The median period between scans was 184 days with a range of 1–2650 days. At the time of the first scan, the median age of this group was 22 years. Twice-scanning of these subjects allowed us to perform scan–rescan correlation analysis to assess the stability of individual differences in the brain anatomy measures described below. In principle, if the first and second scans for given individuals had tended to be performed with the same acquisition protocol (see below), there was potential for scan–rescan correlations to be inflated: however, there were no systematic relations of scans for twice-scanned subjects with respect to heterogeneity of image acquisition.

### Image acquisition

MRI data were acquired with either a 1.5-Tesla Siemens Sonata or Avanto scanner or a 3 Tesla Siemens Trio, TimTrio or Skyra scanner (Siemens Medical Systems, Erlangen, Germany). Given that images were acquired during several smaller scale studies, the parameters used were slight variations of a standard T1-weighted three-dimensional magnetization prepared rapid gradient echo sequence (MPRAGE; 1.0 × 1.0 × 1.0 mm voxel size). The most common variations in the TR/TI/TE/sagittal-slices parameters were the following: 2300/1100/3.03/192, 2730/1000/2.95/176, 2250/850/2.95/176, 2250/850/3.93/176, 2250/850/3.68/176, 2300/1100/3.03/192, 2300/1100/2.92/192, 2300/1100/2.96/192, 2300/1100/2.99/192, 1940/1100/3.93/176 and 1960/1100/4.58/176. To account for magnetic field strength effects, an inhomogeneity correction was applied. There was also variation in the head coils used. The following arrays were employed (with their frequencies) in the right-handed participants: 32-channel (24 %), 12-channel (4 %), 8-channel (38 %) arrays, and single head coil (33 %). In the left-handed participants, this distribution was 32-channel (27 %), 12-channel (0 %), 8-channel (33 %) arrays, and single head coil (40 %).

### Image processing

T1 images were processed using the VBM8 tool and its default settings (http://www.neuro.uni-jena.de/vbm/), implemented in SPM8 (Wellcome Department of Imaging Neuroscience Group, London, UK; http://www.fil.ion.ucl.ac.uk/spm). This procedure segments T1 images into gray matter (GM), white matter (WM), and cerebrospinal fluid (CSF). It then generates the corresponding tissue maps spatially normalized to MNI space (Ashburner 2007) and modulated by the non-linear component of their spatial transformation. The resulting GM images contained information on local volume differences, independent of overall differences in brain size (http://www.neuro.uni-jena.de/vbm/segmentation/modulation/).

In addition, T1 images were independently processed using FreeSurfer’s (v5.3) default “recon-all” pipeline, which performs automated segmentation of non-cortical tissues, as well as automated parcellation of the cerebral cortex (Fischl et al. 2002, 2004).

#### Measurement of regional volumes

Our analyses focused on the cerebellum, and cortical areas corresponding with the classically defined perisylvian language network, i.e., regions of the inferior frontal gyrus and superior temporal gyrus, as well as the post- and precentral gyri due to their involvement in motor cognition and handedness (see “Introduction”). Volumetric estimates of these regions of interest were derived from the processed T1 images in two ways.

**Fig. 1:**
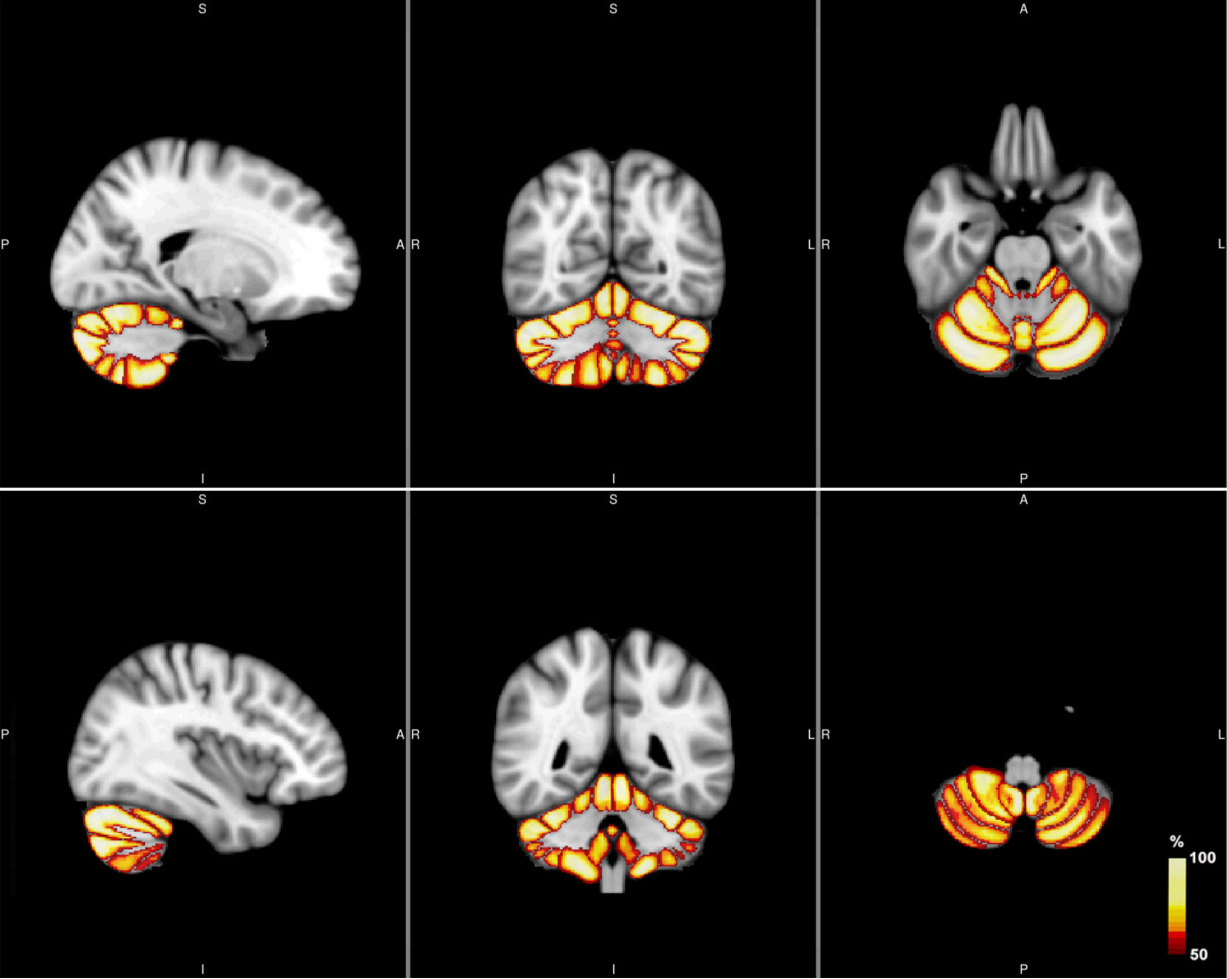
Regional measurement of cerebellar gray matter by the Diedrichsen atlas. The voxels assigned to a given region with 50 % or higher probability are shown (in MNI space). The probabilities are *color coded* (see *bottom right corner*). Coordinates (*X*, *Y*, *Z*) for the *first* and *second rows,* respectively: 70, 65, 47 and 52, 79, 21. *P* posterior, *A* anterior, *S* superior, *I* inferior, *R* right, *L* left

**Fig. 2:**
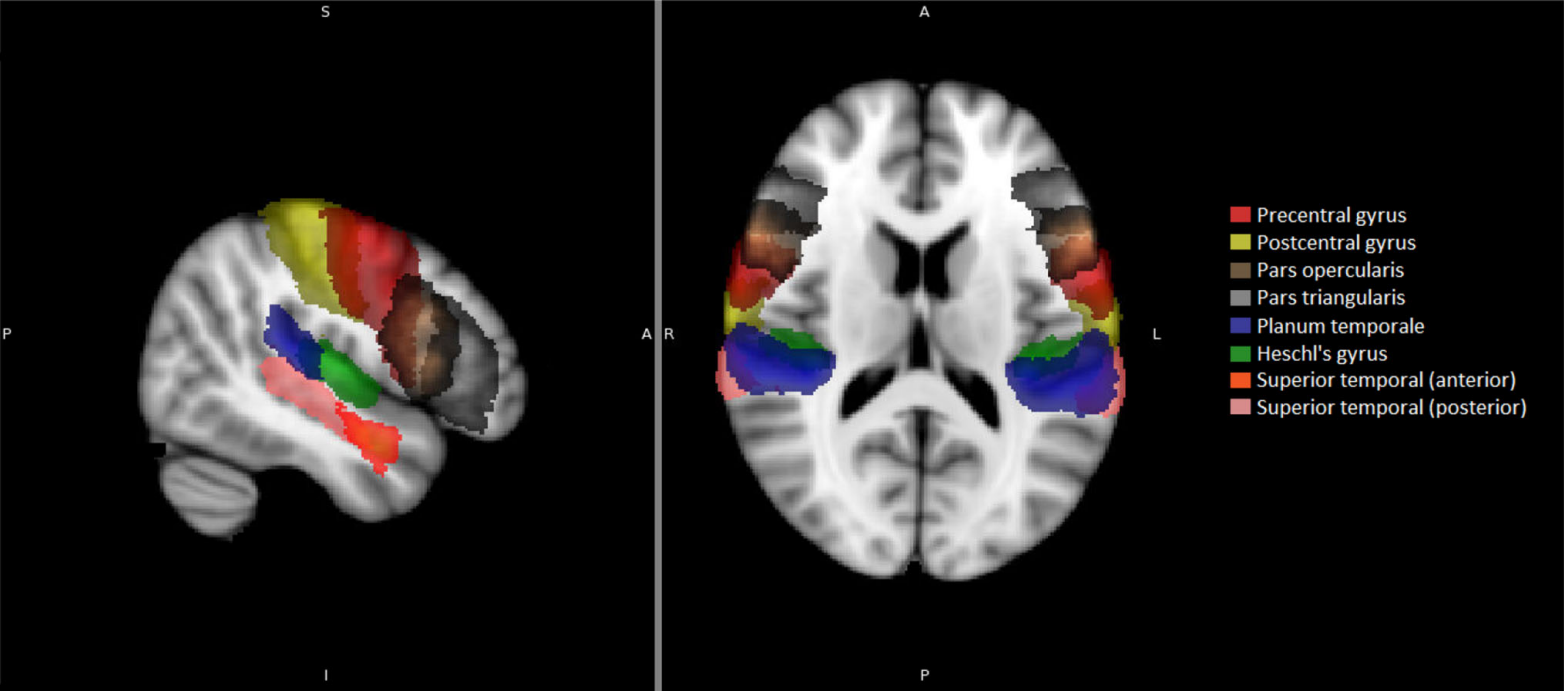
The eight HO-defined cerebral cortical regions selected in this study for analysis of their asymmetry in relation to cerebellar asymmetry

First, regional volumes were extracted from the spatially normalized GM images according to probabilistic atlas definitions. In other words, for a given probabilistic region of interest, we performed a voxel-wise sum of gray matter volumes, weighted by the probability of each voxel belonging to that specific region. Cerebellar estimates were based on the Diedrichsen atlas (Diedrichsen et al. 2009), which contains probabilistic definitions for 28 cerebellar regions in standard space (Fig. 1), 10 of which have left–right counterparts. Only those voxels were included for which the probability weight of belonging to the cerebellum was at least 50 %, to prevent the unintended inclusion of cerebral cortical GM voxels into cerebellar regions. This threshold also meant that cerebellar regions did not generally overlap with each other (see Fig. 1). Cerebral cortical volumes were estimated by the probabilistic Harvard–Oxford (HO) cortical structural atlas that defines 48 bilateral cortical regions in standard space (Goldstein et al. 1999, 2007). Of the 48 bilateral regions, the following was selected and splits at the center of the left–right axis: pars opercularis, pars triangularis, superior temporal gyrus (anterior), superior temporal gyrus (posterior), planum temporale, Heschl’s gyrus, postcentral gyrus, and precentral gyrus (see Fig. 2). Given that there was no overlap between these cortical regions of interest and GM cerebellar voxels, no further manipulation of the HO atlas or of its probabilistic regions was applied. The Diedrichsen and HO atlases were distributed with the FSL software package (http://www.cma.mgh.harvard.edu/fsl_atlas.html).

**Fig. 3:**
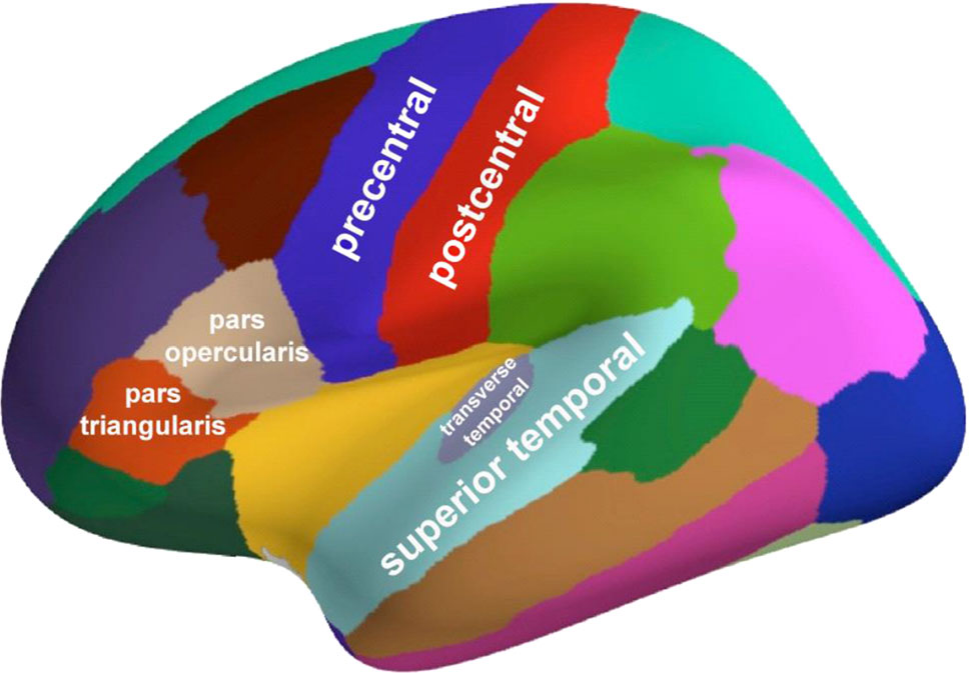
The six Freesurfer-defined cerebral cortical regions (Desikan atlas) selected in the present study for analysis of their asymmetry in relation to cerebellar asymmetry. *Different colors* mark the regions on an inflated brain image

Second, regional cortical volumes were derived from FreeSurfer’s cortical anatomical parcellations, according to the Desikan atlas (Desikan et al. 2006). The selected regions were the pars opercularis, pars triangularis, superior temporal, transverse temporal, precentral, and postcentral cortex (See Fig. 3). FreeSurfer estimates of cerebellar volumes were also derived from its segmentation of the cerebellum into gray and white matters, and further into the left and right structures, but these data were not used further after visual quality control (see below).

#### Quality checks

We visually inspected the spatially normalized GM maps of all study participants, with respect to two main features: the overall quality of the normalized image, and the correct application of the cerebellar probabilistic atlas with regard to non-cerebellar tissue. The spatially normalized GM images were visualized alone and also overlaid with the cerebellar probabilistic atlas, from 35 internal slices of coronal and sagittal views per participant. Images that had not normalized correctly to the standard brain appeared as distorted or incomplete, and were excluded from further analysis. Detailed inspection showed that these problems resulted from overall low image quality, head-motion artifacts, or unusual anatomy. In addition, images were excluded when we detected an overlap between probabilistic cerebellar definitions and wrongfully segmented dura or sinuses. After applying all of these exclusion criteria, the remaining sample size was 2226 (103 left-handers).

Inspection of FreeSurfer’s cortical parcellations was performed independently of the above, again for the entire data set, and followed the protocol developed by the ENIGMA consortium (Thompson et al. 2014) (http://enigma.ini.usc.edu/protocols/imaging-protocols/). Specifically, it consisted of visually checking individual parcellations, plotted from both internal (axial and coronal) as well as external (lateral and medial) views. Individual measurements derived from erroneous parcellations, and in some cases, whole images were excluded from analysis. Erroneous parcellations were identified from internal views when cortical regions were missing, left–right homologous labels were not grossly comparable in position, or cerebral cortical labels had been mapped to non-cortical tissue (e.g., the cerebellum or dura mater). From external views, global errors could be visualized as a rough/spiky brain surface or highly fragmented and interspersed cortical labels. External views also revealed poor anatomical labeling, specifically when the ‘banks of the superior temporal sulcus’ label mapped extensively onto the externally visible brain surface and affected surrounding regions, and when the ‘supramarginal gyrus’ label extended into the superior temporal gyrus. After excluding the data that did not pass these quality filters, all regional measures except for the superior temporal gyrus had a sample size of 2003 (97 left-handers), while for the superior temporal gyrus, the sample size was 1676 (87 left-handers). The overlap of this sample with the quality checked, spatially normalized GM data, was 1875 participants (88 left-handers), for all regions apart from the superior temporal gyrus. For the superior temporal gyrus, the overlap was 1572 participants (79 left-handers).

After the visual quality control, the number of twice-scanned participants with data available for scan–rescan correlation analysis was 329 for the cerebellum and HO cortical data, 277 with Freesurfer data for all cortical regions apart from the superior temporal gyrus, and 226 with Freesurfer data for the superior temporal gyrus.

Freesurfer’s cerebellar segmentations were also visually inspected by plotting them against participants’ scans in a set of axial and coronal views. Focus was placed on detecting segmentation errors with its surrounding dura mater or dural sinuses, as these are complex structures whose intensities on T1 images are particularly similar to those of cerebellar gray matter (Hwang et al. 2011). An initial inspection of 50 random subjects revealed that these problems, although subtle, occurred frequently (>30 % of the visualized subjects). Freesurfer cerebellar measures were subsequently excluded from our analyses.

In addition, each cerebellar and cerebral cortical measure was approximately normally distributed (not shown), and we excluded outlier values beyond plus or minus 3.5 standard deviations (SD) from the mean. Stability of individual difference measurement was assessed by correlating the values for the twice-scanned subjects from the first scan to the second scan, by Pearson’s correlation.

### Asymmetry analysis

For each structure and participant, asymmetry was measured by an asymmetry index (AI) using the formula (*L* − *R*)/(*L* + *R*) where *L* stands for left-side volume and *R* stands for right-side volume. Outlier removal and scan–rescan correlations for AIs were performed as described above (“Quality checks”). Whether the mean AIs differed significantly from zero was tested by *t* tests. All AIs were then adjusted by linear regression (iteratively reweighted least squares) for the potential covariate effects of age, estimated intracranial volume (ICV), sex, field strength, scanner type, and their two-way interactions (with the exception of field strength*scanner type). In addition, we included quadratic terms for age and ICV. All further analyses were conducted using the residuals from these regressions. Not all terms were significant for all AIs, but the inclusion of non-significant terms had negligible effects on the residuals. This uniform approach had the advantage that results could be compared across structures, rather than making them contingent on individual models for each cerebellar lobular AI and cerebral cortical AI.

### Associations with handedness and cerebral cortical asymmetries

Welch’s two sample *t* tests were conducted to assess potential associations between cerebellar AIs and handedness (Welch 1947). This test avoids assumptions of balanced group sizes and equal variances. Pearson’s correlation coefficients were used for assessing the correlations between cerebellar AIs and the AIs of the cerebral cortical regions. Bonferroni correction was applied separately for the correlation analyses of cerebellar AIs with HO-derived cerebral cortical AIs (80 tests) and Freesurfer-derived cerebral cortical AIs (60 tests).

## Results

### Probabilistic atlas for cerebellar lobule gray matter volumes

#### Cerebellar regional gray matter volumes

Table 1 summarizes scan–rescan correlation coefficients for cerebellar regional gray matter volume measures, as well as the median volumes for each scan of the twice-scanned subjects. All measures showed high scan–rescan correlations (greater than 0.75) indicating stable measurement of individual differences.

**Table 1:** Scan–rescan Pearson correlation coefficients for cerebellar regional gray matter volumes (mm^3^), as quantified using the Diedrichsen probabilistic atlas

Cerebellum region	Scan–rescan correlation	Scan median	Rescan median
Left I.IV	0.86	192.68	191.28
Left V	0.88	254.94	255.26
Left VI	0.82	809.68	814.71
Left Crus.I	0.90	1090.63	1089.47
Left Crus.II	0.79	813.50	812.30
Left VIIb	0.78	315.74	312.38
Left VIIIa	0.77	288.05	288.55
Left VIIIb	0.75	197.60	200.53
Left IX	0.84	171.47	174.27
Left X	0.81	21.25	21.54
Right I.IV	0.83	202.44	201.68
Right V	0.84	249.80	249.19
Right VI	0.82	741.55	744.35
Right Crus.I	0.88	1123.91	1106.74
Right Crus.II	0.81	767.73	757.51
Right VIIb	0.79	304.88	299.97
Right VIIIa	0.77	304.32	305.02
Right VIIIb	0.72	228.34	230.13
Right IX	0.78	221.22	222.10
Right X	0.78	24.43	24.64

Data are shown only for cerebellar regions that have the left- and right-sided counterparts defined in this atlas

#### Cerebellar regional gray matter volume AIs

Descriptive statistics for the cerebellar regional AIs are shown in Table 2. Mean AIs for all structures differed significantly from zero (*p* < 0.01). Scan–rescan correlations ranged from 0.48 (region VIIIa) to 0.79 (region I.IV); see Table 2. The correlations were generally higher for the more anterior regions. Only region VIIIa showed a scan–rescan correlation less than 0.5, indicating that most of the measures captured a substantial proportion of stably measured variance across scans.

**Table 2:** Scan–rescan Pearson correlation coefficients for cerebellar regional gray matter AIs, and descriptive statistics of the AIs, as defined by the Diedrichsen probabilistic atlas

Cerebellum region AI	Scan–rescan correlation	Summary statistics				
		Sample size	Mean	SD	Max	Min
I.IV	0.79	2219	−0.03	0.03	0.07	−0.13
V	0.74	2215	0.01	0.02	0.09	−0.07
VI	0.69	2216	0.04	0.02	0.11	−0.04
Crus.I	0.68	2212	−0.01	0.03	0.09	−0.12
Crus.II	0.65	2202	0.03	0.03	0.16	−0.08
VIIb	0.60	2206	0.02	0.04	0.17	−0.13
VIIIa	0.48	2213	−0.02	0.05	0.15	−0.20
VIIIb	0.50	2216	−0.07	0.06	0.15	−0.29
IX	0.57	2215	−0.13	0.03	−0.01	−0.26
X	0.68	2212	−0.07	0.07	0.19	−0.33

Data are only shown for cerebellar regions that have the left- and right-sided counterparts defined in this atlas

### Handedness and cerebellar lobule gray matter asymmetries

Two sample *t* tests, not assuming comparable group sizes, did not reveal significant differences between left- and right-handers in any cerebellar gray matter regional AIs (not shown). The lowest nominal P value (not adjusted for multiple testing) was 0.12 for the AI of region V.

### Analyses of cerebral cortical regions

#### Scan–rescan correlations for left and right volumes

Table 3 shows the scan–rescan correlation coefficients derived from the twice-scanned subjects for each of the selected cerebral cortical volumetric measures in mm^3^, as derived from the HO atlas. The median volumes from each scan of the twice-scanned subjects are also shown. Similarly, Table 4 shows the scan–rescan correlations for cerebral cortical regional volumes derived from Freesurfer. All scan–rescan correlations were >0.8. The generally high correlations indicate a high stability of individual difference measurement, notwithstanding the heterogeneity of scanning parameters.

**Table 3:** Scan–rescan Pearson’s correlation coefficients for regional cerebral cortical gray matter volumes (mm^3^) as defined by the HO atlas

Anatomical measures	Scan–rescan correlation	Scan median	Rescan median
Language-related cortical volumes			
Left pars opercularis	0.90	292.82	290.38
Left pars triangularis	0.84	224.19	220.30
Left superior temporal anterior	0.92	111.66	111.87
Left superior temporal posterior	0.89	188.24	188.18
Left Heschl’s gyrus	0.91	143.61	144.07
Left planum temporale	0.95	262.52	260.78
Right pars opercularis	0.89	273.77	271.42
Right pars triangularis	0.86	222.48	219.79
Right superior temporal anterior	0.92	114.87	112.80
Right superior temporal posterior	0.89	194.03	191.85
Right Heschl’s gyrus	0.90	122.24	120.38
Right planum temporale	0.94	202.07	200.53
Hand motor-related cortical volumes			
Left postcentral	0.80	891.90	883.72
Left precentral	0.82	1132.10	1118.87
Right postcentral	0.80	818.28	800.41
Right precentral	0.81	1124.50	1113.68

**Table 4:** Scan–rescan Pearson’s correlation coefficients for regional cerebral cortical volumes (mm^3^) as defined by Freesurfer

Anatomical measures	Scan–rescan correlation	Scan median	Rescan median
Language-related cortical volumes			
Left pars opercularis	0.94	5475.0	5491.5
Left pars triangularis	0.91	4011.0	3950.0
Left superior temporal	0.93	13285.0	13188.0
Left transverse temporal	0.91	1277.0	1288.0
Right pars opercularis	0.92	4428.0	4435.0
Right pars triangularis	0.90	4711.0	4639.0
Right superior temporal	0.94	12742.0	12625.0
Right transverse temporal	0.91	968.0	970.5
Hand motor-related cortical volumes			
Left postcentral	0.90	10380.0	10261.0
Left precentral	0.90	14260.0	14070.0
Right postcentral	0.92	9629.0	9667.0
Right precentral	0.89	14139.0	13814.0

#### Asymmetry indexes (AIs)

Descriptive statistics for the cerebral cortical regional AIs (including only the first scan values when subjects were scanned twice) are shown in Table 5 (HO) and Table 6 (Freesurfer). The mean AIs for all measures differed significantly from zero (*p* < 0.01). Strong leftward mean asymmetries were measured for two well-known left-lateralized structures: Heschl’s gyrus (i.e., transverse temporal gyrus) and the planum temporale, as well as the pars opercularis (Tables 5, 6). Scan–rescan correlations for AIs are also shown in Tables 5 and 6. All scan–rescan correlations were ≥0.80, indicating robust measurement of individual differences in regional cortical AIs, notwithstanding heterogeneity of scan acquisition.

**Table 5:** Scan–rescan Pearson correlation coefficients and descriptive statistics for HO-derived cerebral cortical AIs

AI	Scan–rescan correlation	Summary statistics				
		*N*	Mean	SD	Max	Min
Language-related cortical AIs						
Pars opercularis	0.90	2213	0.04	0.04	0.19	−0.11
Pars triangularis	0.86	2220	0.00	0.05	0.17	−0.16
Superior temporal anterior	0.92	2223	−0.01	0.06	0.15	−0.20
Superior temporal posterior	0.92	2215	−0.01	0.05	0.14	−0.17
Heschl’s gyrus	0.91	2216	0.09	0.05	0.25	−0.07
Planum temporale	0.96	2219	0.13	0.05	0.30	−0.03
Hand motor-related cortical AIs						
Postcentral	0.85	2218	0.05	0.04	0.17	−0.07
Precentral	0.83	2211	0.00	0.03	0.10	−0.09

**Table 6:** Scan–rescan Pearson correlation coefficients and descriptive statistics for FreeSurfer-derived cerebral cortical AIs

AI	Scan–rescan correlation	Summary statistics				
		Sample size	Mean	SD	Max	Min
Language-related cortical AIs						
Pars opercularis	0.90	1991	0.10	0.08	0.36	−0.18
Pars triangularis	0.87	1995	−0.08	0.08	0.18	−0.36
Superior temporal	0.88	1671	0.02	0.04	0.16	−0.12
Transverse temporal	0.80	1991	0.13	0.09	0.38	−0.17
Hand motor-related cortical AIs						
Postcentral	0.89	1996	0.03	0.05	0.22	−0.15
Precentral	0.84	1989	0.01	0.04	0.14	−0.13

### Cerebellar regional gray matter asymmetries and cerebral cortical asymmetries

Table 7 shows the correlations between cerebellar regional gray matter AIs and cerebral cortical regional AIs as measured using the HO atlas. These correlations were all low, ranging from *r* = −0.08 to *r* = 0.14. In total, 11 cerebellar-HO AI correlations were significant at alpha 0.05, after multiple testing correction over all cerebellar-HO AI tests (80 tests). Most of these correlations were positive, indicating ipsilateral rather than contralateral correlation. The lowest nominal (uncorrected) P values were 8E−11 for the correlation between the AI of cerebellar region I.IV and the AI of Heschl’s gyrus (*r* = 0.14), and *P* = 5E−10 for the correlation between the AI of cerebellar region I.IV and the AI of the planum temporale (*r* = 0.13). Heschl’s gyrus in the HO atlas is comparable with FreeSurfer’s ‘transverse temporal’ region, whose AI also showed a very low correlation with that of cerebellar region I.IV (Table 8: *r* = 0.05, uncorrected *P* = 0.05), consistent in direction for the HO- and Freesurfer-defined region.

**Table 7:** Pearson correlation coefficients between cerebellar regional gray matter AIs and cerebral cortical regional AIs as derived from the HO atlas

Cerebellar regional AI	Language-related AIs												Hand motor-related AIs			
	Pars opercularis		Pars triangularis		Superior temporal anterior		Superior temporal posterior		Planum temporale		Heschl’s gyrus		Postcentral		Precentral	
	Cor	*p*	Cor	*p*	Cor	*p*	Cor	*p*	Cor	*p*	Cor	*p*	Cor	*p*	Cor	*p*
I.IV	−0.02	0.39	−0.03	0.22	−0.03	0.23	0.03	0.11	**0.13**	**5E−10**	**0.14**	**8E−11**	−0.04	0.08	−0.01	0.78
V	−0.03	0.13	−0.02	0.32	**−0.08**	**1E−04**	−0.03	0.12	0.05	0.01	0.05	0.02	−0.04	0.10	0.07	2E−03
VI	0.03	0.11	0.02	0.29	**−0.08**	**3E−04**	**−0.08**	**4E−04**	0.01	0.78	−0.02	0.48	0.02	0.46	**0.08**	**1E−04**
Crus.I	**0.08**	**3E−04**	0.05	0.01	−0.06	5E−03	−0.05	0.03	−0.01	0.63	−0.03	0.13	0.05	0.02	**0.09**	**3E−05**
Crus.II	0.04	0.09	0.02	0.39	**0.09**	**2E−05**	**0.08**	**2E−04**	0.06	0.01	0.05	0.03	**0.13**	**4E−09**	0.00	0.96
VIIb	0.01	0.81	0.01	0.66	−0.03	0.14	−0.02	0.42	−0.03	0.10	−0.02	0.28	0.06	5E−03	0.04	0.05
VIIIa	0.02	0.36	0.01	0.62	−0.02	0.45	−0.04	0.06	0.00	0.95	0.04	0.08	0.03	0.14	0.05	0.03
VIIIb	0.03	0.17	0.03	0.17	−0.02	0.32	−0.01	0.52	0.02	0.26	0.04	0.09	0.03	0.17	0.01	0.79
IX	0.01	0.76	0.02	0.46	0.04	0.09	0.01	0.64	0.00	0.83	0.02	0.34	0.06	4E−03	0.02	0.46
X	0.03	0.16	−0.03	0.18	−0.02	0.37	0.02	0.47	0.05	0.03	0.02	0.45	−0.03	0.22	0.02	0.37

Correlations are shown in bold font when significant (alpha 0.05) after Bonferroni correction for 80 tests

**Table 8:** Pearson correlation coefficients (cor) and nominal *p* values (*p*) between cerebellar regional gray matter AIs and cerebral cortical regional AIs as derived by FreeSurfer

Cerebellar regional AI	Language-related AIs								Hand motor-related AIs			
	Pars opercularis		Pars triangularis		Superior temporal		Transverse temporal		Postcentral		Precentral	
	Cor	*p*	Cor	*p*	Cor	*p*	Cor	*p*	Cor	*p*	Cor	*p*
I.IV	−0.02	0.44	0.02	0.32	−0.05	0.07	0.05	0.05	0.00	0.96	0.02	0.31
V	0.01	0.58	0.00	0.96	**−0.09**	**2E−04**	−0.01	0.78	0.01	0.60	0.05	0.02
VI	0.04	0.09	−0.01	0.62	−0.07	0.01	−0.03	0.22	0.01	0.62	0.03	0.23
Crus.I	0.02	0.35	0.02	0.36	−0.04	0.15	−0.06	0.01	0.01	0.64	−0.01	0.53
Crus.II	−0.02	0.51	−0.06	0.01	0.07	0.01	0.02	0.38	0.02	0.40	0.02	0.34
VIIb	0.05	0.03	−0.01	0.72	0.02	0.53	0.00	0.88	0.01	0.75	0.00	0.88
VIIIa	0.04	0.10	0.04	0.12	0.02	0.44	0.00	1.00	0.03	0.18	−0.03	0.16
VIIIb	0.01	0.79	0.04	0.07	−0.01	0.57	0.00	0.84	0.01	0.68	−0.02	0.47
IX	0.04	0.07	0.00	0.91	−0.01	0.65	−0.03	0.26	0.03	0.26	−0.01	0.70
X	0.02	0.49	0.00	0.87	−0.01	0.79	0.02	0.51	−0.07	0.00	0.00	0.96

Only one correlation, shown in bold font, was significant at alpha 0.05 after Bonferroni correction for 60 tests

Table 8 shows all of the correlations between cerebellar regional gray matter AIs and cerebral cortical regional AIs as derived from Freesurfer. Only one correlation (*r* = −0.09, uncorrected *P* = 2E−04) was significant at alpha 0.05 after multiple testing correction over all cerebellar-Freesurfer cortical tests (60 tests), which was for the AI of cerebellar region V with the AI of the ‘superior temporal’ region. Stricter correction for multiple testing (e.g., over all 80 cerebellar-HO and 60 cerebellar-Freesurfer tests) would render this result insignificant. This finding for cerebellar region V was consistent in direction with the HO AI for the anterior superior temporal gyrus (*r* = −0.08, uncorrected *P* = 2E−04).

The hand motor-related cortical regional AIs showed no correlations with cerebellar AIs which were consistent across both HO and Freesurfer, and significant after multiple testing correction.

## Discussion

Lateralization of cerebellar activation and connectivity has been previously reported in relation to motor and language tasks. However, little was known about how these properties may be reflected in terms of brain anatomy. Here, we investigated individual differences in left–right volumetric cerebellar asymmetries in a data set comprising 2226 healthy individuals, in relation to cerebral cortical asymmetries of regions involved in either motor control or language, and also with respect to handedness. We used automated methods for quantifying asymmetries of cerebellar and cerebral cortical regional volumes, together with extensive visual quality control. Ours was by far the largest study of cerebellar anatomical asymmetries to have been performed.

In this large study, there was no evidence for relationships between individual differences in cerebellar asymmetries and handedness. Some significant correlations of cerebellar regional asymmetries to cerebral cortical asymmetries were found, including for the asymmetry of cerebellar region I.IV with the asymmetry of Heschl’s gyrus, although none of these correlations were greater than 0.14. A correlation of 0.14 indicates that only 2 % of variance is shared between measures. Furthermore, these weak correlations were mostly not robust across methods/atlases, and they were predominantly ipsilateral rather than contralateral in nature. Our results, therefore, form a clear contrast to the previous literature on strong, contralateral cerebellar–cerebral activations related to hand motor control and language cognition (see Introduction), and underscore once more that links between structural and functional lateralization in the human brain are extremely complex and indirect (Greve et al. 2013).

We used linear regression to adjust for major scanning differences, including non-linear interaction terms, before testing associations with handedness or cerebral cortical anatomy. It remains possible that some aspects of image acquisition heterogeneity were not completely corrected by this procedure, and therefore, that subtle biases induced by scan heterogeneity may have given rise to weak and spurious associations in the data. Some of the significant but weak cerebellar–cerebral cortical correlations of asymmetry that we found may have been due to this. Alternatively, the weak correlations that we found may represent true biological relations between cerebellar and cerebral cortical anatomical asymmetries, although they have no predictive value from cerebellum to cortex or vice versa. Their validity will need to be investigated in additional datasets. Regardless, it is clear that our data indicated no overt associations of cerebellar asymmetries to handedness or cerebral cortical lateralization.

Although our data set included a degree of heterogeneity in terms of scanning parameters used across participants, we used scan–rescan correlations in over 200 twice-scanned subjects to assess how stably the individual differences were measured in spite of this heterogeneity. As there were no overt relations of handedness or scan–rescan participation to specific acquisition protocols, the stability of measurement indicated by the scan–rescan correlations can be taken as a fair reflection of measurement robustness, given the heterogeneity in acquisition. Most unilateral volumetric measures and AIs showed scan–rescan correlations that were high enough to indicate substantial proportions of variance being due to stably measured individual differences. However, the scan–rescan correlation for the asymmetries of some of the posterior cerebellar regional gray matter asymmetries showed relatively low scan–rescan correlations. The low stability for these latter measures of asymmetry might have partially masked any possible associations with handedness and cerebral cortical asymmetries, insofar as low scan–rescan correlations are likely to be indicative of measurement error. However, since we found no substantial associations with handedness or cerebral cortical asymmetries when testing the cerebellar asymmetries that had high stability of measurement, we consider it unlikely that cerebellar anatomical asymmetry is overtly linked to these aspects of brain and behavioral asymmetry.

The probabilistic gray matter atlas that we used divides the cerebellum into lobules, but it is still possible that finer-resolution asymmetries, found within the lobules, may relate anatomically to cerebral cortical lateralization and/or handedness, to a greater extent than we found in the present study. This may be possible given that activations with contrasting lateralizations have been reported for certain sub segments within lobules (Wang et al. 2013). Future anatomical studies may, therefore, benefit from voxel-wise comparisons (Buckner et al. 2011). In addition, there may be asymmetries involving the cerebellar vermis which were not possible for us to detect, given that the method used in our study did not differentiate the vermis into left and right.

We only tested correlations between cerebellar asymmetries and selected language- or motor-related cerebral cortical regions which were likely candidates for showing structure–function links in lateralization. Cerebellar projections to elsewhere in the cortex might, however, also contribute to language-related or motor functions (Buckner et al. 2011; Buckner 2013). Indeed, there are established connections between certain cerebellar lobules and cortical association areas, especially of the prefrontal cortex (Bostan et al. 2013), which can motivate future studies of additional cerebral cortical regions.

It is interesting that handedness showed no relation to cerebellar asymmetrical anatomy, given that hand motor actions map to cerebellar regions with a high degree of precision (Mottolese et al. 2013; van der Zwaag et al. 2013). From a developmental perspective, it is noteworthy that at 10 weeks of gestation most human embryos move their right arms more than their left arms (Hepper et al. 1998), while motor asymmetries at 15 weeks gestation have been shown to predict handedness in children that were followed longitudinally (Hepper et al. 2005). These early motor asymmetries in utero may reflect neural asymmetries relatively caudally in the CNS (e.g., spinal cord and brain stem), since connections of the arms with forebrain structures are still poorly developed or absent (Hepper et al. 1998). The hindbrain and spinal cord may even be important developmental origins of asymmetry in the human CNS that precede cerebral cortical lateralization, particularly with respect to hand preference. As a hindbrain structure, the adult cerebellum might, therefore, have been expected to vary with handedness in its anatomy. As we saw no relation of cerebellar asymmetry to handedness, then presumably if the embryonic hindbrain is involved in setting up brain asymmetry related to hand preference, it may occur at a stage before the cerebellum itself has differentiated within the hindbrain, or else only continues to manifest in adulthood in terms of functional asymmetry.

An important issue with respect to handedness is how exactly to define the trait. Although multi-item questionnaires are often used with respect to hand preference for sets of manual actions, it has been shown that simple self-assessments of overall handedness, such as that used in the present study (asking subjects only to categorize themselves as the left- or right-handed) show close agreement with dichotomous scoring of handedness as derived from multi-item inventories, as well as robust test–retest repeatability (Bryden et al. 1991; Ransil and Schachter 1994; Tan 1993). We are, therefore, confident of the validity of the binary, self-reported assessment of handedness that was used in our study. Although the group sizes of left- and right-handers included in our analysis were not comparable, our statistical method of testing the group difference was robust to this (Ruxton 2006). In addition, there was no systematic difference in scanning parameters applied for left- and right-handers.

The atlases used to define brain regions in this study contained asymmetrical definitions for all structures that were asymmetrical, on average, in the reference data sets originally used to create those atlases. Accordingly, the measurement of mean asymmetry indexes in our own data set would inevitably reflect left–right differences present in the atlases. For detecting cerebral cortical asymmetries with automated methods, some groups have chosen to work from artificially created, left–right symmetrical atlases (Kawasaki et al. 2008). However, our study was focused on comparing relative degrees of asymmetry between subjects and groups, i.e., using the individual and group-level differences in AIs, regardless of the mean population level of asymmetry. The use of ‘real-world’ asymmetrical atlases, rather than artificially symmetrized atlases, was, therefore, appropriate for our study, as it had the advantage that regional identification was likely to be more accurate for structures that were asymmetrical both in the atlases and, on average, in our data set. We did not aim to measure absolute levels of asymmetry, nor confirm mean population-level asymmetry of any of the regions under study.

Brain asymmetries are relatively subtle aspects of human anatomy and physiology. Our study highlights the utility of studying brain asymmetries in large data sets of thousands of subjects, using automated measurement, to achieve definitive information on the relationships, or lack of relationships, between asymmetries in different brain regions, and factors that may affect them such as handedness.
